# Differentiating amnestic from non-amnestic mild cognitive impairment subtypes using graph theoretical measures of electroencephalography

**DOI:** 10.1038/s41598-022-10322-9

**Published:** 2022-04-13

**Authors:** Jae-Gyum Kim, Hayom Kim, Jihyeon Hwang, Sung Hoon Kang, Chan-Nyoung Lee, JunHyuk Woo, Chanjin Kim, Kyungreem Han, Jung Bin Kim, Kun-Woo Park

**Affiliations:** 1 Department of Neurology, Korea University Anam Hospital, Korea University College of Medicine, Seoul, Republic of Korea; 2 Department of Neurology, Korea University Guro Hospital, Korea University College of Medicine, Seoul, Republic of Korea; 3 Laboratory of Computational Neurophysics, Brain Science Institute, Korea Institute of Science and Technology, Seoul, Republic of Korea

**Keywords:** Cognitive ageing, Cognitive neuroscience, Computational neuroscience, Diseases of the nervous system

## Abstract

The purpose of this study was to explore different patterns of functional networks between amnestic mild cognitive impairment (aMCI) and non-aMCI (naMCI) using electroencephalography (EEG) graph theoretical analysis. The data of 197 drug-naïve individuals who complained cognitive impairment were reviewed. Resting-state EEG data was acquired. Graph analyses were performed and compared between aMCI and naMCI, as well as between early and late aMCI. Correlation analyses were conducted between the graph measures and neuropsychological test results. Machine learning algorithms were applied to determine whether the EEG graph measures could be used to distinguish aMCI from naMCI. Compared to naMCI, aMCI showed higher modularity in the beta band and lower radius in the gamma band. Modularity was negatively correlated with scores on the semantic fluency test, and the radius in the gamma band was positively correlated with visual memory, phonemic, and semantic fluency tests. The naïve Bayes algorithm classified aMCI and naMCI with 89% accuracy. Late aMCI showed inefficient and segregated network properties compared to early aMCI. Graph measures could differentiate aMCI from naMCI, suggesting that these measures might be considered as predictive markers for progression to Alzheimer’s dementia in patients with MCI.

## Introduction

Mild cognitive impairment (MCI) has been considered as a transitional cognitive state between normal aging and dementia^1^. There is growing interest in MCI as the drugs currently available to treat Alzheimer's disease (AD) can only suppress symptoms of dementia for a limited period, they cannot stop or reverse disease progression^2^. MCI is a heterogeneous group with diverse prognosis, where some progress to dementia, remain as MCI, or recover to normal cognition; therefore, classification of MCI is important for predicting outcomes and establishing treatment strategies^3^. MCI can be categorized into several subtypes based on the number of impaired domains (single- vs. multi-domain) and impairment of the memory domain (amnestic vs. non-amnestic)^1,4^.

Among the subtypes of MCI, the amnestic form of MCI (aMCI) is more likely to convert to AD dementia than other subtypes of MCI and healthy elders^3,5^; thus, aMCI has been regarded as a precursor of AD dementia. Several lines of evidence suggest that treatment with acetylcholine esterase inhibitors may delay progression to AD in patients with aMCI^6,7^. Given the possibility of attempting therapeutic interventions, early detection of aMCI before conversion to AD is critical in the management of patients with cognitive decline. Moreover, classification of aMCI into early and late stages may provide insight into underlying etiology, pathophysiology, and prognosis, which could ultimately provide important information for establishing a treatment strategy^8,9^.

It is widely accepted that cognitive dysfunction in AD could be attributed to a functional disconnection between distant brain areas^10,11^. Integration of neural activities between different brain regions is required for physiological brain functioning; therefore, analyzing the disruption of functional connectivity (FC) between brain regions may provide more information regarding pathophysiological mechanisms than investigating the activities of individual brain regions in AD. Various neuropathological, electrophysiological, and neuroimaging studies have provided evidence that AD disrupts the integration of FCs between brain regions, leading to cognitive decline^10,12–15^. Furthermore, a FC analysis using resting-state electroencephalography (EEG) showed an altered neural coupling in MCI as well as AD, and the degree of alteration was greater in AD than MCI^15^. These findings suggest that FC indices could be a useful biomarker to quantitatively evaluate abnormalities in the MCI-AD continuum^15^. Although identifying distinct patterns of FC in each subtype of MCI might provide important information for differentiating etiologies and predicting prognosis^16^, there is a paucity of studies comparing FC properties between subtypes of MCI^17^.

Recent advances in graph theoretical network analysis enable the assessment of the topological architecture of complex human brain networks^18,19^. Therefore, graph theoretical analysis could be an optimal framework for quantitatively characterizing network properties in each subtype of MCI. A recent study showed disintegrated resting-state network properties in patients with aMCI relative to controls by applying graph theoretical network analysis^20^. In addition, another EEG graph analysis revealed that aMCI small-world architecture presents midway topological properties between healthy controls and AD, supporting the hypothesis that aMCI is a precursor of AD dementia^21,22^. To the best of our knowledge, there is no EEG study applying graph theoretical analysis to explore distinct patterns of FC in each subtype of MCI. Herein, we aimed to compare the FC properties between aMCI and non-aMCI (naMCI) to explore the distinct patterns of aMCI networks and evaluate the usefulness of EEG graph measures for differentiating aMCI from naMCI using machine learning algorithms without comprehensive neuropsychological tests. We hypothesized that aMCI might have less integrated FC than naMCI and that late aMCI might have much less integrated FC than early aMCI. We also hypothesized that using graph measures reflecting network integration and segregation may enable discrimination between aMCI and naMCI with high accuracy.

## Methods and materials

### Participants

**Figure 1:**
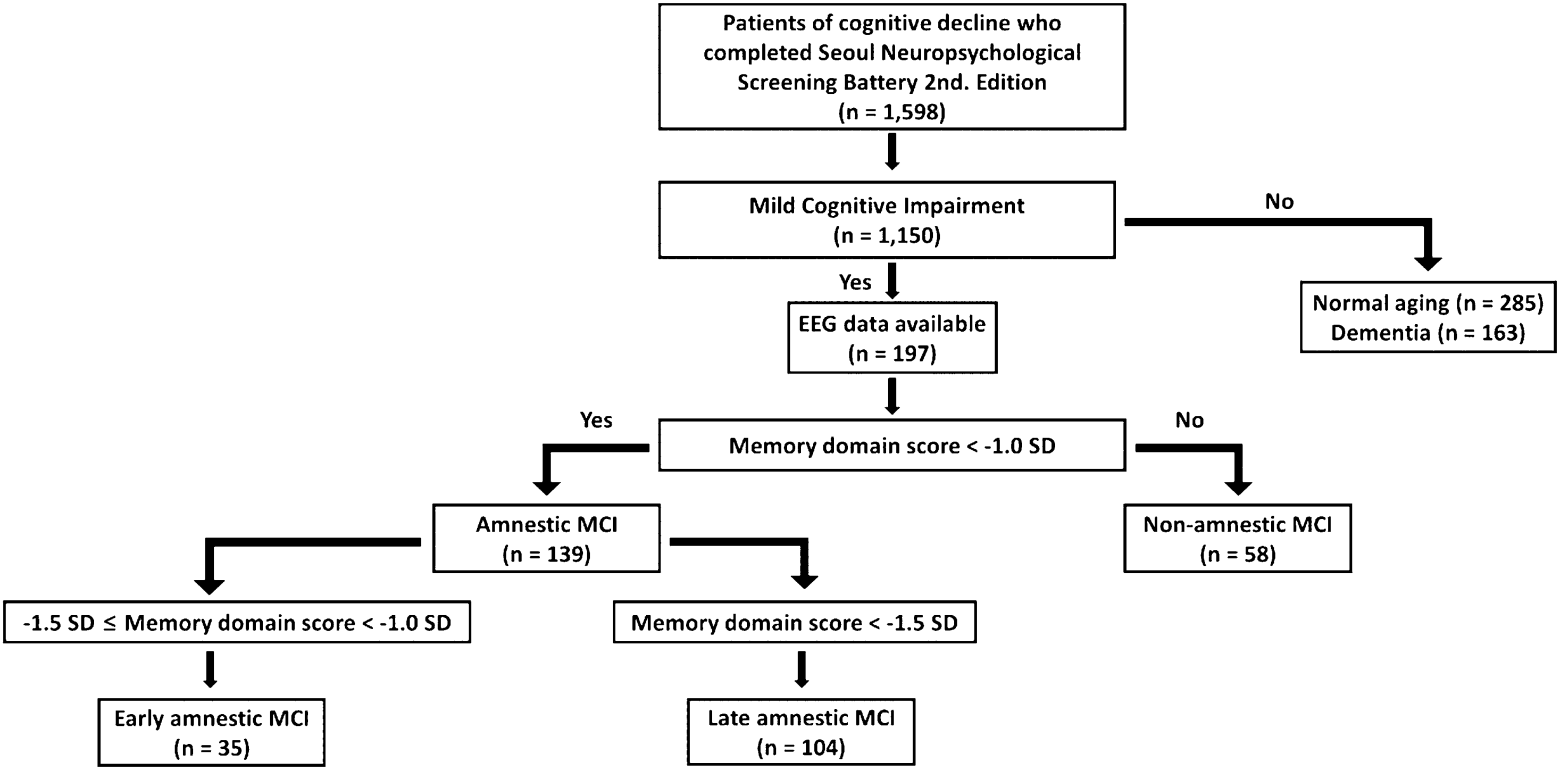
A flow chart of participant classification.

A dataset of neuropsychological tests from 1598 drug-naïve individuals who complained of cognitive impairment was reviewed. Among the individuals, 197 patients with MCI who completed the EEG recordings were included in this study. MCI was operationally defined as follows: (1) subjective memory complaints by the patient and/or caregiver; (2) objective cognitive impairment below − 1.0 SD in one or more cognitive domains assessed using comprehensive neuropsychological test; (3) no significant impairment in activities of daily living; and (4) no dementia^23,24^. Compared to the age and education norms, MCI patients with below − 1.0 SD in-memory domain scores were classified as aMCI, and otherwise were classified as naMCI. Within the aMCI, patients with memory domain scores in the range of − 1.5 SD to − 1.0 SD below norms were classified as early aMCI, and those with < − 1.5 SD below norms were classified as late aMCI^4,9^. A flow chart of the participant classification is shown in Fig. 1. The study followed the ethical guidelines of the Declaration of Helsinki and was approved by the local ethics committee of the Korea University Anam Hospital (No. 2021AN0021). Informed consent was obtained from all individual participants included in the study.

### Neuropsychological tests

The Mini-Mental State Examination (MMSE) was used to assess global neurocognitive function. All participants underwent neuropsychological testing using the Seoul Neuropsychological Screening Battery second edition (SNSB-II)^25^. We chose to use eight cognitive measures, which were representative and important neuropsychological tests to evaluate five cognitive domains as follows: (1) Memory: the Seoul Verbal Learning Test (SVLT) delayed recall (verbal memory) and Rey-Osterrieth Complex Figure Test (RCFT) delayed recall (visual memory); (2) Language: Korean version of the Boston Naming Test (K-BNT); (3) Visuospatial function: RCFT copying Test; (4) Frontal executive function: tasks for animal names and supermarket items and a phonemic portion of the Controlled Oral Word Association Test (COWAT) and the Stroop Test (color reading); and (5) Attention: Digit Span Test forward and backward. Results with numeric and continuous values were used in the analysis.

### EEG recording

The EEG examination was performed for 30 min using a 32-channel recording system (COMET plus, Grass Technologies Inc., West Warwick, RI, USA) with 19 scalp electrodes (Fp1, Fp2, F7, F8, F3, F4, T3, T4, C3, C4, T5, T6, P3, P4, O1, O2, Fz, Cz, and Pz) placed according to the international 10–20 system. EEG data were sampled at 200 Hz, and the bandpass filter was set between 0.1 and 70 Hz. Ten non-consecutive resting-state 2-s epochs for each participant were carefully reviewed and selected by two board-certified neurologists according to the following protocol: (1) presence of continuous physiological alpha activity with voltage maximum in posterior regions; (2) absence of artifacts, epileptiform discharges, and other nonstationary elements; and (3) absence of patterns indicating drowsiness or arousal. Resting-state EEG data were used for the analysis of the functional networks in this study.

### Graph theoretical and statistical analyses

Resting-state FC was evaluated by coherence, which reflects the level of functional signal communication between different regions of the brain^26^. Coherence was defined as $COH_{xy} = k_{xy}^{2} \left( f \right) = \left| {K_{xy} \left( f \right)} \right|^{2} = \frac{{\left| {S_{xy} \left( f \right)} \right|^{2} }}{{S_{xx} \left( f \right)S_{yy} \left( f \right)}}$, where *S*_*xy*_*(f)* is the cross-spectral density between *x* and *y*, and *Sxx(f)* and *Syy(f)* are the auto-spectral densities of *x* and *y*, respectively. where *K* represents the coherency function. |*S*| denotes the modulus of *S*. The coherence value ranged between 0 and 1, with 0 denoting no statistical relationship and 1 being full coherence^26^. The epochs were then bandpass filtered into the following frequency bands: delta (0.1–4 Hz), theta (4–8 Hz), alpha (8–13 Hz), beta (13–30 Hz), and gamma (30–50 Hz). The subsequent analyses were performed separately for each band. Network properties were characterized using a weighted undirected network model of graph-theoretic analysis to avoid the arbitrariness of threshold selection and preserve the continuous nature of the correlated information^27^. Graph measures (average degree, average strength, radius, diameter, characteristic path length, global efficiency, local efficiency, clustering coefficient, transitivity, modularity, assortativity, and small-worldness) were computed using the Brain Connectivity Toolbox (http://www.brain-connectivity-toolbox.net) and BRAPH toolbox (http://braph.org) working on MATLAB R2019b (MathWorks, Natick, MA, USA)^27,28^. Graph measures were compared between aMCI and naMCI, as well as between early aMCI and late aMCI groups using non-parametric tests with 5000 permutations. Statistical significance was set at *P* < 0.05 and corrected for multiple comparisons using the false discovery rate (FDR). Differences in the neuropsychological test results between the groups were compared using an independent *t*-test. Correlation between the neuropsychological test results and graph measures found to be discriminant between aMCI and naMCI was evaluated using Pearson’s correlation analysis (*P* < 0.05).

### Machine learning applications

Two EEG graph measures (i.e., modularity in beta band and radius in gamma band) which manifested significant differences between aMCI and naMCI groups were chosen for the input features for machine learning algorithms. In addition, two essential clinical information (i.e., sex and MMSE total score) were also used as input features. Since we aimed to determine whether aMCI could be screened based on resting-state EEG-based parameters that can be obtained without comprehensive neuropsychological tests reproducibly, the features related to SNSB-II were not considered in the machine learning tasks. Approaches of feature selection for applying machine learning algorithms are described in our previous studies^29,30^. To evaluate the utility of the selected input features to discriminate the two groups (i.e., aMCI and naMCI), six different traditional machine learning methods were implemented using Python’s Orange toolbox (v.3.29.3)^31^: logistic regression, support vector machine (SVM)^32^, random forest classifiers^33^, gradient boosting, neural network, and naïve Bayes^34^. Six EEG graph measures (i.e., degree, strength, global efficiency, local efficiency, clustering coefficient, and transitivity in gamma band) which manifested significant differences between early aMCI and late aMCI groups were chosen for the input features for machine learning algorithms. The above-mentioned machine learning algorithms were applied to distinguish between early aMCI and late aMCI. The classification of machine learning methods was validated using a random sampling algorithm with 70% of the data used for training and balance for testing. A stratified random sampling strategy was used, and the procedure was repeated 10 times. The performance of each classifier was evaluated using a confusion matrix that contains the parameters of precision, recall, accuracy, and F1 score, as follows:

$$Precision = \frac{TP}{{\left( {TP + FP} \right)}}$$

$$Recall = \frac{TP}{{\left( {TP + FN} \right)}}$$

$$Accuracy = \frac{{\left( {TP + TN} \right)}}{{\left( {TP + TN + FP + FN} \right)}}$$

$$F1 \;score = \frac{2 \times precision \times recall}{{precision + recall}}$$

where TP, FP, TN, and FN refer to the number of true positives, false positives, true negatives, and false negatives, respectively. Recall and specificity were computed to generate receiver operating characteristics. The area under the curve for receiver operating characteristics was also computed.

## Results

### Demographic characteristics and neuropsychological tests

The differences in demographic characteristics and neuropsychological tests between the groups are shown in Table 1. The proportion of women was lower in the aMCI group than in the naMCI group. The performance in neuropsychological tests, which included MMSE total score, SVLT delayed recall, RCFT delayed recall, K-BNT, RCFT copy, COWAT animal, and supermarket, and Stroop test was poorer in the aMCI group than in the naMCI group (all *P* < 0.05). The duration of education was shorter in the early aMCI group than in the late aMCI group. There was no difference in the performance of neuropsychological tests, except SVLT delayed recall and RCFT delayed recall, between the early and late aMCI groups.

**Table 1:** Demographic characteristics and results of neuropsychological tests.

	aMCI (*n* = 139)	naMCI (*n* = 58)	*P*	Early aMCI (*n* = 35)	Late aMCI (*n* = 104)	*P*
Age (years)	73.83 ± 9.10	74.03 ± 7.05	0.877	74.40 ± 7.61	73.63 ± 9.57	0.668
Female sex (n, %)	65 (46.76)	41 (70.69)	**0.002**	16 (45.71)	49 (47.11)	0.886
Education years	9.03 ± 5.16	7.52 ± 4.56	0.054	7.31 ± 5.60	9.61 ± 4.90	**0.023**
MMSE total score	23.95 ± 3.83	26.03 ± 3.01	** < 0.001**	23.69 ± 4.13	24.04 ± 3.75	0.639
SVLT delayed recall	− 1.63 ± 0.92	0.00 ± 0.77	** < 0.001**	− 0.76 ± 0.76	− 1.93 ± 0.77	** < 0.001**
RCFT delayed recall	− 1.27 ± 0.94	0.01 ± 0.68	** < 0.001**	− 0.77 ± 0.93	− 1.44 ± 0.88	** < 0.001**
DST forward	0.85 ± 1.02	0.99 ± 0.98	0.372	0.88 ± 0.92	0.84 ± 1.04	0.856
DST backward	− 0.36 ± 1.28	− 0.12 ± 1.16	0.224	− 0.62 ± 1.21	− 0.27 ± 1.30	0.168
K-BNT	− 0.59 ± 1.87	0.22 ± 1.33	**0.001**	− 0.35 ± 1.54	− 0.67 ± 1.97	0.373
RCFT copy	− 0.85 ± 1.81	− 0.05 ± 0.94	** < 0.001**	− 0.61 ± 1.11	− 0.94 ± 1.99	0.356
COWAT animal	− 0.76 ± 1.09	− 0.08 ± 1.19	** < 0.001**	− 0.54 ± 0.94	− 0.84 ± 1.13	0.160
COWAT supermarket	− 0.67 ± 0.84	0.14 ± 1.08	** < 0.001**	− 0.50 ± 0.75	− 0.73 ± 0.87	0.175
COWAT phonemic	− 0.83 ± 1.04	− 0.58 ± 0.91	0.108	− 0.71 ± 0.88	− 0.86 ± 1.09	0.475
Stroop test	− 0.92 ± 1.53	− 0.19 ± 1.09	**0.001**	− 1.11 ± 1.79	− 0.86 ± 1.44	0.417

*aMCI* amnestic mild cognitive impairment, *naMCI* non-amnestic mild cognitive impairment, *MMSE* Mini-Mental State Examination, *SVLT* Seoul Verbal Learning Test, *RCFT* Rey-Osterrieth Complex Figure Test, *DST* Digit Span Test, *K-BNT* the Korean version of the Boston Naming Test, *COWAT* controlled oral word association test.

Significant values are in [bold].

### Graph theoretical analyses

**Figure 2:**
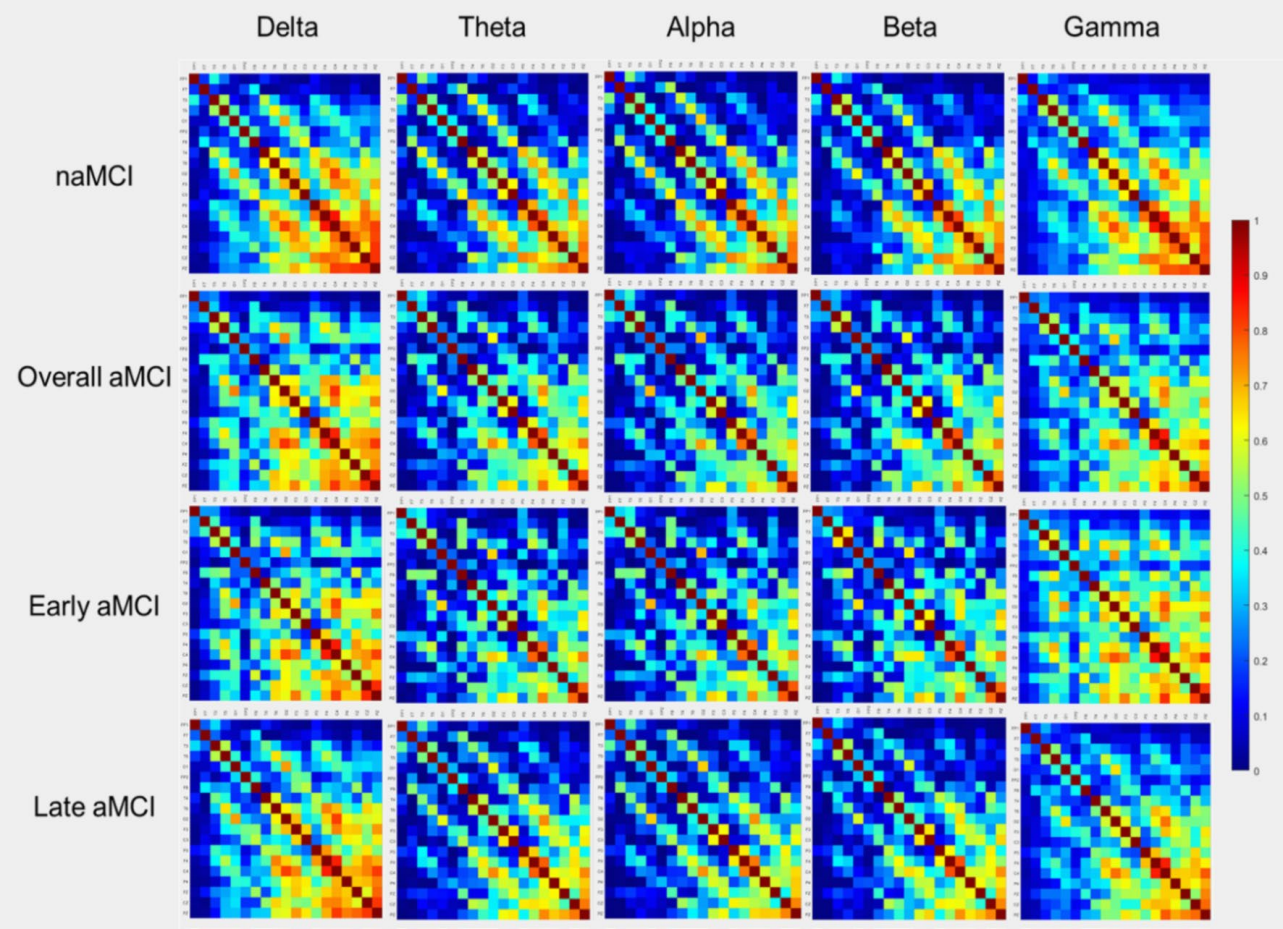
Adjacency matrices of coherence. The plots show the coherence between 19 pairs of scalp electroencephalography electrodes in each frequency band in non-amnestic mild cognitive impairment (naMCI), overall amnestic mild cognitive impairment (aMCI), early aMCI, and late aMCI.

FC in terms of coherence is represented by the adjacent matrices in Fig. 2. Comparisons of global graph measures between the aMCI and naMCI groups are shown in Table 2. Compared to naMCI, higher modularity in the beta band and lower radius in the gamma band were found in aMCI (FDR-corrected *P* < 0.05). Comparisons of global graph measures between early and late aMCI are presented in Table 3. Compared to early aMCI, late aMCI showed lower degree, strength, global efficiency, local efficiency, clustering coefficient, and transitivity in the gamma band (FDR-corrected *P* < 0.05).

**Table 2:** Comparisons of graph measures between aMCI and naMCI.

Graph measures	Delta		Theta		Alpha		Beta		Gamma	
	aMCI	naMCI	aMCI	naMCI	aMCI	naMCI	aMCI	naMCI	aMCI	naMCI
Degree	12.402	12.278	10.414	10.307	9.956	10.285	10.219	10.664	12.448	12.465
Strength	6.599	6.500	4.849	4.877	4.429	4.634	4.527	4.837	6.153	6.414
Radius	12.674	8.417	18.963	10.654	5.711	6.253	7.116	7.228	9.186*	16.029*
Diameter	17.759	13.517	23.131	14.770	9.772	10.504	12.068	12.408	16.620	23.787
Characteristic path length	4.193	3.846	5.104	4.225	3.838	3.898	4.221	4.082	4.415	5.588
Global efficiency	0.443	0.444	0.383	0.384	0.365	0.375	0.361	0.372	0.417	0.428
Local efficiency	1.088	1.081	0.793	0.809	0.718	0.747	0.714	0.760	0.970	1.047
Clustering coefficient	0.408	0.404	0.323	0.328	0.301	0.305	0.297	0.312	0.366	0.380
Transitivity	0.666	0.648	0.511	0.515	0.463	0.475	0.480	0.506	0.588	0.618
Modularity	0.140	0.154	0.241	0.257	0.277	0.260	0.242*	0.219*	0.156	0.141
Assortativity	0.145	0.153	0.218	0.209	0.212	0.203	0.286	0.275	0.136	0.125
Small-worldness	0.865	0.883	1.022	0.868	0.894	0.908	0.875	0.892	0.890	0.839

*aMCI* amnestic mild cognitive impairment, *naMCI* non-amnestic mild cognitive impairment.

Bold font with an asterisk (*) represents statistical significance (false discovery rate-corrected *P* < 0.05).

**Table 3:** Comparisons of graph measures between early aMCI and late aMCI.

Graph measures	Delta		Theta		Alpha		Beta		Gamma	
	Early aMCI	Late aMCI	Early aMCI	Late aMCI	Early aMCI	Late aMCI	Early aMCI	Late aMCI	Early aMCI	Late aMCI
Degree	12.126	12.320	10.129	10.298	10.229	10.008	10.854	10.199	13.296*	12.093*
Strength	6.335	6.475	4.723	4.830	4.634	4.434	4.815	4.535	7.263*	5.846*
Radius	6.306	14.743	12.471	6.276	6.867	5.719	7.433	7.302	12.196	12.503
Diameter	11.293	20.038	16.588	10.444	11.078	9.843	12.191	12.576	20.690	20.466
Characteristic path length	3.481	4.570	4.419	3.800	4.020	3.868	4.007	4.321	4.787	5.153
Global efficiency	0.435	0.440	0.379	0.382	0.376	0.364	0.373	0.358	0.470*	0.401*
Local efficiency	1.042	1.068	0.777	0.800	0.753	0.715	0.746	0.714	1.215*	0.918*
Clustering coefficient	0.397	0.401	0.321	0.327	0.307	0.299	0.302	0.300	0.423*	0.351*
Transitivity	0.643	0.647	0.501	0.512	0.471	0.462	0.481	0.488	0.669*	0.572*
Modularity	0.153	0.153	0.268	0.250	0.271	0.274	0.242	0.234	0.135	0.158
Assortativity	0.162	0.143	0.219	0.209	0.183	0.210	0.259	0.284	0.101	0.142
Small-worldness	0.978	0.841	0.855	0.868	0.875	0.894	0.887	0.900	0.832	1.088

*aMCI* amnestic mild cognitive impairment.

Bold font with an asterisk (*) represents statistical significance (false discovery rate-corrected *P* < 0.05).

### Correlation analyses

**Figure 3:**
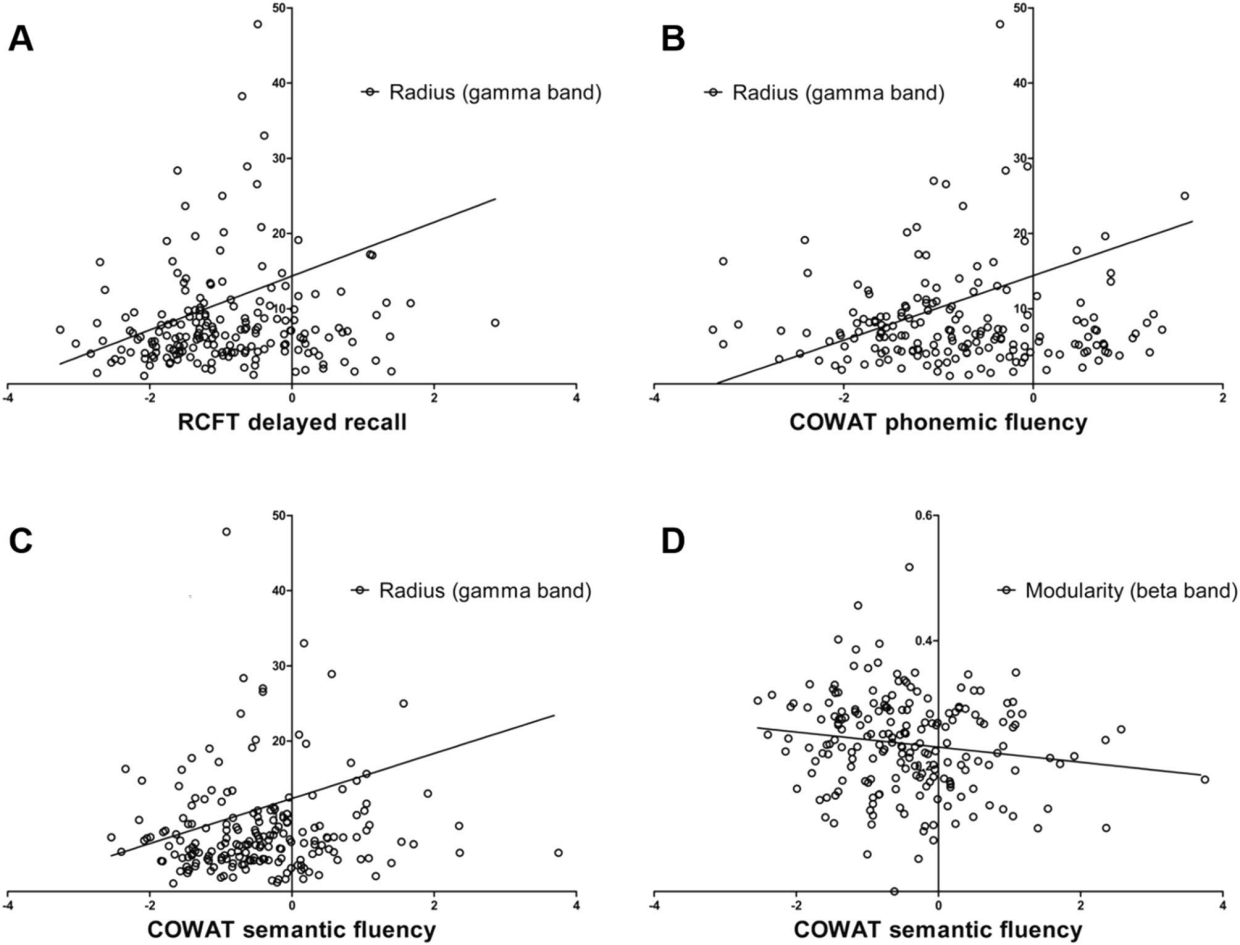
Results of correlation analyses.

The radius in the gamma band was positively correlated with RCFT delayed recall (*r* = 0.208, *P* = 0.003), COWAT phonemic fluency (*r* = 0.236, *P* = 0.001), and COWAT semantic fluency (*r* = 0.164, *P* = 0.021; Fig. 3A–C). Modularity in the beta band was negatively correlated with COWAT semantic (supermarket items) fluency (*r* = − 0.155, *P* = 0.030; Fig. 3D). There was no relationship between the results of other neuropsychological tests and the graph measures found to be discriminant between aMCI and naMCI (radius in gamma band and modularity in beta band).

### Machine learning applications

**Figure 4:**
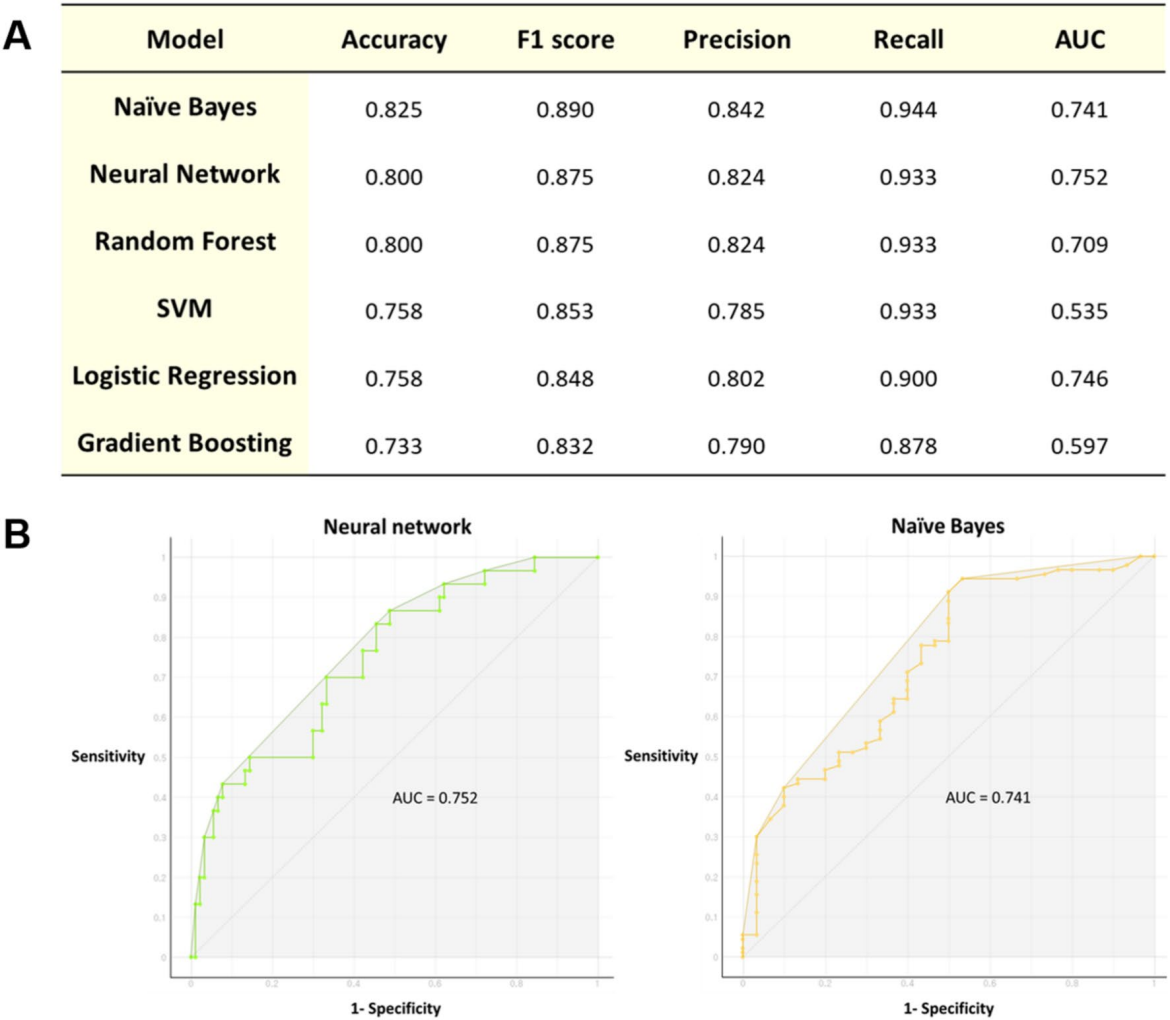
Performance of machine learning algorithms for classification between aMCI and naMCI. The receiver operating characteristic (ROC) curve of the naïve Bayes classifier is plotted (left panel). F1 scores in each machine learning algorithm are presented in the right panel. Abbreviations: AUC, the area under the curve; SVM, support vector machine.

The four variables (sex, MMSE total score, modularity in the beta band, and radius in gamma band) found to be possible predictors of aMCI in group comparisons were selected for use as input features in machine learning algorithms. The performances of the machine learning algorithms are presented in Fig. 4. The F1 scores for classification between aMCI and naMCI were 0.890 for naïve Bayes, 0.875 for the random forest, 0.875 for neural network, 0.853 for SVM, 0.848 for logistic regression, and 0.832 for gradient boosting. The six variables (degree, strength, global efficiency, local efficiency, clustering coefficient, and transitivity in gamma band) found to be different measures in group comparisons between early aMCI and late aMCI were selected for use as input features in machine learning algorithms. The F1 scores for classification between early aMCI and late aMCI were 0.872 for SVM and logistic regression, 0.865 for neural network, 0.788 for random forest, 0.776 for gradient boosting, and 0.661 for naïve Bayes.

## Discussion

We investigated differences in resting-state EEG FC between aMCI and naMCI patients using graph theoretical analysis. We found that patients with aMCI showed higher modularity in the beta band and lower radius in the gamma band relative to those with naMCI. Modularity in the beta band was associated with lower COWAT semantic fluency test, and radius in the gamma band was correlated with higher RCFT delayed recall, COWAT phonemic fluency, and COWAT semantic fluency tests. The naïve Bayes algorithm with modularity in the beta band, radius in the gamma band, sex, and MMSE total score as input features could classify aMCI and naMCI with 89% accuracy. In subgroup analysis, patients with late aMCI showed lower average degree, average strength, global efficiency, local efficiency, clustering coefficient, and transitivity in the gamma band than those with early aMCI. The SVM and logistic regression with the six graph measures could classify early aMCI and late aMCI with 87% accuracy.

Modularity is a global graph measure of network segregation^35,36^, calculated by partitioning a network into groups of modules with high connectivity within modules relative to the connectivity between regions in distinct modules^37,38^. Thus, our finding of higher modularity in aMCI relative to naMCI suggests that the network property of aMCI may be more segregated than that of naMCI. Eccentricity refers to the maximal distance between a certain node and any other node, and radius represents the minimum eccentricity of all nodes in the network^27,28^. Since dense networks have many connections between the nodes in the network, the eccentricity would be higher than that of disconnected or sparse networks. Therefore, we speculate that our finding of a lower radius in aMCI might be attributed to sparse long-distance FC. Collectively, our findings of graph measures suggest that patients with aMCI have segregated networks.

It is generally accepted that flexible reconfiguration across brain regions and networks is required for complex cognitive processing^18,39^. Converging findings from multimodal neuroimaging studies suggested that disrupted integration in large-scale brain networks may be critical pathophysiological mechanisms underlying AD and that these segregations might be responsible for the cognitive deficits^40–42^. In an EEG graph theoretical analysis, characteristic path length was found to be longer in beta band in patients with AD relative to healthy controls, and the longer path length was associated with the lower MMSE scores for all participants^13^. Our findings of inefficient and less integrated network property found in aMCI relative to naMCI are similar to those of AD in the aforementioned studies. Additionally, our findings of late aMCI with much less integrated networks than early aMCI suggest that the degree of network segregation may be associated with the severity of cognitive deficits, which could support the notion that early aMCI, late aMCI, and AD are on the spectrum of cognitive decline.

The relationships between the graph measures and cognitive function tests reflecting domains other than memory, particularly frontal executive function, imply that resting-state EEG-based graph measures could sensitively detect the predisposition of cognitive decline and predict the possibility of transition to dementia with impairments of two or more cognitive domains. A recent longitudinal study showed that aMCI patients with frontal executive dysfunction had a higher risk of dementia conversion than those with visuospatial or language dysfunction, supporting our speculation^43^. Several lines of evidence suggest that indices from FC analysis of EEG could be considered as a marker for the diagnosis of AD or cognitive decline^13,44,45^. A significant decrease in EEG synchrony in alpha and beta bands was observed in patients with AD compared with healthy controls and MCI, and the loss of synchronization in the beta band was found to be associated with lower cognitive scores^44,45^. An EEG graph theoretical analysis found that the characteristic path length in the beta band was significantly longer in patients with AD than in controls^13^. Although each study used different indices to evaluate FC, these findings commonly suggest that altered FC in the high-frequency band might be implicated in the pathophysiology of AD and cognitive decline AD pathophysiology and cognitive decline. Our finding of segregated network property in late aMCI was found mainly in the gamma band, which may be in line with previous studies suggesting the important role of high-frequency synchronization in cognitive processing^46,47^.

We observed that machine learning algorithms using the four parameters (modularity in beta band, radius in gamma band, sex, and MMSE total score) as input features were highly accurate in differentiating aMCI from naMCI. Considering that neuropsychological tests require a relatively long time to perform, machine learning algorithms using features based on short-term resting-state EEG might be considered as convenient screening tools for differentiating aMCI from naMCI. Future prospective studies with large populations are required to verify the reliability of the application of machine learning algorithms for detecting aMCI.

Our study has several limitations. First, this was a cross-sectional study; therefore, the value of graph theoretical measures for predicting prognosis is speculative. Second, our study was based on the population of patients who were referred to the single university-affiliated hospital; therefore, it may be difficult to apply our findings to the general population. Third, although evaluation of the AD-specific biomarker has become important in classifying MCI and diagnosing AD spectrum disease, we could not evaluate the amyloid status in the present study. Finally, since the normal aging population was not included in this study, we could not understand the network changes in the spectrum from normal aging through aMCI to AD. Nevertheless, this is the first study to apply resting-state EEG network analysis to differentiate aMCI from naMCI in a relatively large population. Notably, it is the strength of our study that all patients were included in the drug-naïve state, which allowed us to exclude drug effects on the EEG results. Moreover, the results of a machine learning classifier that discriminated aMCI and naMCI with relatively high accuracy using EEG graph measures without the results of comprehensive neuropsychological tests may provide insight into the application of machine learning methods using EEG indices in the diagnosis of aMCI to establish a treatment strategy to prevent conversion to AD.

## Conclusion

We found that the functional network of aMCI is segregated relative to that of naMCI by applying resting-state EEG FC analysis for the first time, and the properties of aMCI are similar to those of the previously reported AD network. Based on our findings, graph theoretical measures, particularly modularity and radius in high-frequency bands, could differentiate aMCI from naMCI, might be considered as predictive markers for progression to AD in patients with MCI. Since the findings were derived from EEG data of a short period, our results should be interpreted with caution. Further longitudinal studies with large populations and using data of more prolonged period are required to validate the accuracy of predicting AD conversion in MCI using graph theoretical measures. Moreover, further studies with large populations are needed to determine whether the machine learning algorithms could discriminate early aMCI from late aMCI, which may provide additional insights into the usefulness of machine learning applications for classifying the spectrum of cognitive decline in AD.
